# Construction and expression of recombinant human elastin with biological activities: effect of wound healing *in vitro* and *in vivo*

**DOI:** 10.3389/fbioe.2026.1913985

**Published:** 2026-09-10

**Authors:** Xiaona Zhang, Enfang Zhou, Baixin Liao, Yiting Wang, Can Xiong, Mingming Jin, Pei Qin, Sheng Xiong

**Affiliations:** 1 Institute of Biomedicine and Department of Cell Biology, College of Life Science and Technology, Jinan University, Guangzhou, China; 2 National Engineering Research Center of Genetic Medicine, Guangdong Provincial Key Laboratory of Bioengineering Medicine, Jinan University, Guangzhou, China; 3 Guangzhou Cheer-Derm Biotech Co., Ltd., Guangzhou, China; 4 Shandong Freda Biotechnology Co., Ltd., Jinan, China

**Keywords:** elastin, prokaryotic expression system, skin, skin regeneration, wound repair

## Abstract

Elastin (ELN) is a key extracellular matrix protein that is crucial for the elasticity of many vertebrate tissues. Due to the low solubility of the natural elastin, its application in research has been limited, and soluble elastin derivatives has a wider range of applications. At present, the use of prokaryotic expression systems to obtain recombinant protein has the advantages of low cost, controllable sequence, and low immunogenicity, making it the optimal method for large-scale protein production. To address existing limitations, in this work, we designed and constructed two recombinant human elastins, which were expressed, purified, and then evaluated for activity through cell proliferation, migration and antioxidant assays. The EL1 and RE1 significantly promoted the proliferation and migration of 3T3 cells. Studies on a mouse skin wound model demonstrated that two recombinant elastins likely promotes wound healing by mitigating inflammatory responses, accelerating collagen deposition, and regulating CD31 and α-SMA factors to enhance angiogenesis. This study demonstrates two new recombinant human elastins with good biological activity and can promote wound healing in mice, highlighting its potential as a promising biomolecular therapeutic strategy for skin wound repair.

## 1 Introduction

The skin is the first natural barrier to protect human tissues and organs from physical, chemical, and other attacks. Skin injuries are inevitable in human daily life, especially extensive full-thickness injuries caused by accidents and fires, which are difficult to heal and have become a major clinical problem worldwide (Veith et al., 2019). Wound healing is a complex process that includes continuous and overlapping stages, such as hemostasis, inflammation, angiogenesis, growth, re-epithelialization, and remodeling (Rodrigues et al., 2019; Wen et al., 2020; Peña and Martin, 2024). Neutrophils and macrophages are both innate immune cells and play a crucial role in the inflammation and tissue growth stages (Zhu et al., 2021; Raw et al., 2022; de Oliveira et al., 2016). When the skin is damaged by external factors, during the growth of new skin tissue, the regeneration rate of elastin (ELN) is far lower than that of collagen. As a result, the newly formed skin tissue often lacks elasticity and may even produce severe scars (Frech and Lichtenberger, 2024).

ELN is a vital protein component of the extracellular matrix, bestowing elasticity upon tissues and playing a pivotal role in their recovery from deformation and in stretching and contracting movements (Vindin et al., 2019; Baumann et al., 2021). In tissues with high demands for elasticity, the content of elastin is also relatively higher. For instance, in ligaments, elastin accounts for 80% of the protein content, in the lungs it ranges from 10% to 25%, and in the skin, it makes up 3% (Uitto et al., 1991). Although elastin constitutes only 3% of the skin’s protein content, it is vital for maintaining the skin’s morphology and function (Weihermann et al., 2017). Research indicates that elastin and its derivatives can accelerate the healing of chronic wounds and the restoration of the skin barrier function, supplementation with exogenous elastin can reduce the formation of wound scars (Mora et al., 2016; Gardeazabal and Izeta, 2024; Li et al., 2023). Moreover, Elastin also possesses chemotactic properties, capable of inducing cell proliferation and regulating cell differentiation, which are essential for maintaining tissue homeostasis (Vindin et al., 2019; Sakai et al., 1986; Rodgers and Weiss, 2005; Isenburg et al., 2006). The degradation of elastin is often accompanied by the loss of tissue elasticity and aging. Abnormalities in elastin within the body can trigger a series of diseases.

The assembly process of elastin is highly complex (Ozsvar et al., 2021), its precursor (tropoelastin) can assemble under physiological conditions and is subsequently cross-linked to form mature elastic fibers (Zhu et al., 2025; Depenveiller et al., 2024). Its highly cross-linked structure endows it with considerable stability (Vrhovski and Weiss, 1998; Hedtke et al., 2019), a half-life of up to 70 years (Shapiro et al., 1991), and good resistance to enzymatic degradation. In humans, elastin is expressed from the ELN gene. Tropoelastin is the direct translational product of the ELN gene, and dominates the physical performance of human elastic tissue as it is assembled to make elastin (Mithieux et al., 2013). Due to the low solubility of natural elastin, its application in research is limited. However, soluble elastin derivatives, including elastin-like peptides (ELPs), tropoelastin have shown greater potential for application (Depenveiller et al., 2024). At present, harnessing prokaryotic expression systems to produce recombinant elastin offers several advantages, such as low cost, controllable sequence, and low immunogenicity, which has attracted widespread attention (Carlos Rodríguez-Cabello et al., 2005; Li et al., 2024). Through literature research (Bataille et al., 2016; Kakinoki and Yamaoka, 2014; Yao et al., 2004), we selected and constructed two sequences. Based on ELPs, by incrementally inserting (VPGKG) into (VPGIG) repetitive polypeptides, we designed a new sequence EL1. The other recombinant elastin segment (RE1) is constructed by the key structural domains of human elastin sequence. This study aims to design, construct, express, purify, and screen new recombinant elastin, providing new ideas to address the current issues of recombinant elastin design sequences that cannot be expressed and low bio-mimicry. Additionally, the newly prepared recombinant human elastin will undergo *in vitro* activity identification and *in vivo* activity testing using a mouse wound model, laying the foundation for subsequent medical materials related to skin repair.

## 2 Materials and methods

### 2.1 Construction and expression of recombinant human elastin

The DNA sequences encoding EL1 and RE1 were synthesized by sangon biotech company (Shanghai, China), and the pET-28a (+)/EL1 and pET-28a (+)/RE1 plasmids were constructed. Based on the multiple cloning sites of the pET-28a (+) vector, primers were designed (Supplementary Table S1), and the SUMO gene was fused with the EL1 and RE1 genes using overlap PCR to construct the SUMO-EL1 and SUMO-RE1 fusion genes. These fusion genes were then inserted into the pET-28a (+) vector, with the restriction sites Nde I and BamH I chosen for SUMO-EL1, and Nde I and Hind III for SUMO-RE1.

To express EL1 and RE1, the plasmids were transformed into *Escherichia coli* BL21 (DE3) as the expression host. The transformed bacteria were cultured in LB medium (10 g/L tryptone, 10 g/L sodium chloride, and 5 g/L yeast extract) containing 100 μg/mL kanamycin and shaken at 37 °C, 220 rpm. The preliminary trial parameters were set, when the OD600 reached approximately 0.6–0.8, 1 mM IPTG (Sigma, Germany) was added to the medium, and the culture was induced and incubated at 37 °C for 10 h. The bacteria were collected by centrifugation at 5,000 rpm for 10 min and sonicated in a 20 mM Tris-HCl buffer (pH 8.0, 0.15 M NaCl). The soluble extract was centrifuged at 13,000 rpm for 30 min, and protein expression and solubility were analyzed using SDS-PAGE with Coomassie Brilliant Blue staining and Western blot.

### 2.2 SDS-PAGE and western blot

All samples were mixed with a 5×SDS-PAGE loading buffer (Solarbio, China) at a ratio of 4:1 and boiled for 10 min. The proteins were separated using 12% SDS-PAGE prepared in laboratory, and gels were stained with Coomassie Brilliant Blue rapid staining solution (Solarbio, China). The position of protein could be determined based on the molecular weight indicated by the protein marker (ThermoFisher Scientific, United States). The soluble expression level of recombinant fusion protein was analyzed by using a gel imaging system (Bio-Rad, United States). For Western blot, a SDS-PAGE gel was transferred to NC nitrocellulose membrane (Merck, Germany), the molecular weight and expression of recombinant fusion protein were confirmed by western blot using the horseradish peroxidase (HRP) conjugate of anti-6*His tag antibody (Proteintech, China).

### 2.3 Fermentation condition optimization

To optimize the culture conditions of 1 L shake flask through single factor experiments, and the optimization content included expression temperature (16 °C, 23 °C, 30 °C and 37 °C), IPTG concentration (0.1 mM, 0.2 mM, 0.5 mM, 1 mM), expression time after IPTG addition. 10 mL of LB medium was inoculated with *Escherichia coli* and cultured overnight at 37 °C, 220 rpm, and then transferred to 500 mL TB medium for further culture. The bacterial strains were collected by centrifuging, and ultrasonically broken to release the protein. SDS-PAGE was used to detect the expression of recombinant protein with the change of expression temperature, IPTG concentration and IPTG induction time.

### 2.4 Purification of SUMO-EL1 and SUMO-RE1 fusion protein

The bacterial cells were collected, resuspended in a 20 mM Tris-HCl buffer (pH 8.0, 0.15 M NaCl), and subjected to ultrasonic fragmentation on the ice. The supernatant obtained after centrifugation was filtered through a 0.45 µm membrane and then purified using a Ni column (chromatography column specifications: 2.6 × 200 mm, flow rate 2–5 mL/min, column pressure not exceeding 0.2 MPa; or 50 × 400 mm, flow rate 5–10 mL/min, column pressure not exceeding 0.2 MPa). After loading the sample, the column was washed with NTA-0 buffer (20 mM Tris-HCl, pH 8.0 + 0.15 M NaCl) until the baseline was reached. The impurity protein was washed away using NTA-60 buffer (20 mM Tris-HCl, pH 8.0 + 0.15 M NaCl + 60 mM imidazole), and the target protein was eluted with NTA-250 buffer (20 mM Tris-HCl, pH 8.0 + 0.15 M NaCl + 250 mM imidazole). After elution, the protein concentration was measured using a BCA protein assay kit (ThermoFisher Scientific, United States), and the purity of the purified protein was assessed by SDS-PAGE electrophoresis.

### 2.5 Purification of recombinant EL1 and RE1

Different mass ratios (enzyme: protein) of 1: 100, 1: 500, 1: 1000, 1: 2000, 1: 3000, and 1: 4000 were subjected to enzymatic cleavage at 4 °C for 2 h. The cleavage products were then analyzed by SDS-PAGE to evaluate the enzymatic cleavage efficiency. The optimal mass ratio, determined based on the cleavage results, was selected as the enzymatic cleavage condition for SUMO-RE1 and SUMO-EL1. Then, the enzymatically digested protein solution was exchanged to the NTA-0 buffer solution using dialysis. Ni-NTA affinity chromatography was performed using a Ni column again. Prior to purification, 5 mL of Ni resin was loaded into the AKTA avant protein purification system (Cytiva, China), and the Ni column was equilibrated with Ni-column equilibration buffer (20 mM Tris-HCl, pH 8.0, 0.15 M NaCl). The fusion protein after enzymatic hydrolysis was filtered through a 0.45 μm membrane and then loaded onto the Ni column. The purity of the purified protein was assessed by SDS-PAGE.

### 2.6 Cell culture

3T3 cells were incubated in Dulbecco’s modified Eagle’s medium (DMEM, Gibco, United States) supplemented with 10% fetal bovine serum (FBS, Gibco, United States) and 1% penicillin/streptomycin (PS, Gibco, United States) at 37 °C under 5% CO_2_ atmosphere.

### 2.7 Cell proliferation assay

Cells were plated in 96-well plates (5,000 cells/well) and cultured in 37 °C under 5% CO_2_ atmosphere for 24 h. Cells were cultured in DMEM without FBS for 2 h before treating. Different concentrations of EL1 and RE1 dissolved by using DMEM with 2% FBS, with five replicate wells per group. After incubation for 24 h, 10 μL CCK-8 solution (Solarbio, China) was added to each well, followed by incubation for 2 h. The absorbance at 450 nm was measured using a microplate reader (ThermoFisher Scientific, United States).

### 2.8 Scratch assay

Cells were plated in 6-well plates (1 × 10^6^ cells/well), with three replicate wells per group, and cultured for 24 h. A straight line was scratched in each well using a 200 μL pipette tip, and the dislodged cells were gently washed away with PBS. Then, 2 mL of serum-free medium was added to each well, and the cells were starved in the incubator for 2 h. After removing the medium, the wells were treated with pre-diluted solutions: DMEM (0.2% FBS), DMEM (0.2% FBS, 12.5 μg/mL EL1), and DMEM (0.2% FBS, 25 μg/mL RE1). After adding 2 mL to each well, the plates were placed in the cell culture incubator for incubation. The scratch healing process was observed and photographed under a microscope at 12 and 24 h, and the scratch area was quantified using ImageJ software.

### 2.9 Antioxidant activity assay

Cells were plated in 96-well plates (8000 cells/well) and incubated in a CO_2_ incubator for 24 h. 3 mM H_2_O_2_ solution was prepared using DMEM with 0.2% FBS as the diluent, and this solution was further used to prepare different concentrations of elastin proteins EL1 and RE1. Then, 100 μL of each solution was added to the wells to treat the cells for 24 h.

### 2.10 Animal experiments

All animal experiments were approved by the Laboratory Animal Ethics Committee of Jinan University (Approval No. IACUC-20250911-08). SPF grade male BALB/c mice (6–8 weeks old, 20–25 g weight) were purchased from Beijing Vital River Laboratory Animal Technology Co., Ltd. (Beijing, China). The purchased mice were raised for a week to adapt to the environment and then tested. The back hair of the mice was shaved using an electric hair shaver, and the remaining short hair was removed with a depilatory cream. The next day after depilation, the mice were randomly divided into four groups (n = 10): the negative control group (1×PBS), EL1 group, RE1 group, the positive control group (compound polymyxin B ointment). Each time we administered the drug, we apply the fixed dose directly onto the wound: PBS (100 μL/wound area), EL1 (12.5 μg/mL, 100 μL, 1.25 μg/wound area), RE1 (25 μg/mL, 100 μL, 2.5 μg/wound area), compound polymyxin B ointment (∼20 mg/wound area). Then, it was covered with sterile gauze and fixed with medical tape. All local administration doses were standardized based on the wound area to eliminate the interference of individual body weight differences on the local exposure volume. The drug was administered continuously for 1 week. Mice were randomly selected on days 0, 3, 7 and 12, and photographed for subsequent statistics on the changes of the wound area of the skin. The skin tissues were fixed in 4% paraformaldehyde for subsequent experiments after the randomly selected mice were killed on day 3, 7 and 12 respectively.

### 2.11 Histological analysis

The skin tissues collected were fixed in 4% paraformaldehyde for 24 h and embedded in paraffin. The skin sections (5 μm) were stained with hematoxylin-eosin (HE, Servicebio, China) and Masson’s Trichrome to evaluate the healing of skin and collagen deposition in the wound area. All sections were captured by microscopy (IX53, Olympus, Japan) and quantitative collagen deposition analysis was performed by ImageJ software.

### 2.12 Immunofluorescence staining

The prepared paraffin sections were dewaxed and put into citric acid buffer, and the antigens were repaired by microwave oven. Sections were then blocked in 5% bovine serum albumin (BSA, Solarbio, China) for 30 min and incubated overnight with anti-CD68 antibody (1:1000, Servicebio, China), anti-CD31 antibody (1:400, Servicebio, China), anti-α-SMA antibody (1:300, Servicebio, China) and anti-CD206 antibody (1:200, Proteintech, China) at 4 °C. The fluorescent secondary antibody (Alexa Fluor 594, 1:300, Servicebio, China) was incubated for 1 h at room temperature in the dark. Nuclei were counterstained with DAPI (10 μg/mL; Solarbio, China) for 10 min. Finally, the sections were cover-slipped with the anti-fluorescence quenching sealing agent. Fluorescence images were taken with a confocal fluorescence microscope (Leica, Germany) and for quantitative analysis, each Image is analyzed by ImageJ software (NIH, Bethesda, MD, United States).

### 2.13 Statistical analysis

All values are presented as mean ± standard deviation (SD). The statistical analysis was evaluated with Student’s t-test or one-way ANOVA using GraphPad 9.0. In all statistical analyses, p-value less than 0.05 was considered statistically significant, p-values were defined as *<0.05, **<0.01, ***<0.001, and ****<0.0001.

## 3 Results

### 3.1 Expression of the recombinant elastin EL1 and RE1

The theoretical relative molecular weights of EL1 and RE1 are 17.7 kDa and 31.8 kDa, respectively, while the theoretical relative molecular weight of the SUMO tag is 14 kDa. Therefore, the theoretical relative molecular weights of SUMO-EL1 and SUMO-RE1 are 31.7 kDa and 45.8 kDa, respectively. As shown in Supplementary Figure S1, the EL1, SUMO-EL1 and SUMO-RE1 designed in this study can be soluble expressed, and no detectable expression of protein RE1 was observed. However, the expression level of EL1 was relatively low. This indicates that the expression of ELN was rather challenging. From a sequence perspective, EL1 is composed of simple VPGXG sequence repeats, with a highly simplified structure. This simplicity makes it relatively easier to express in *Escherichia coli* compared to RE1, but it also means that it lacks the intricate structures found in natural proteins that are responsible for complex biological functions. The RE1 sequence is derived from the complex human elastin sequence, and its expression in *Escherichia coli* often faces challenges, which is likely the reason for the low production of RE1. The gray-scale scanning was conducted using the ImageJ software, the analysis showed that the percentage of the fusion protein SUMO-EL1 in the total bacterial protein was approximately 35.3%, which was 27.7% higher than the percentage of protein EL1 in the total bacterial protein (7.6%). And SUMO-RE1 could be successfully expressed and exhibited soluble expression under 37 °C induction conditions. After adding the SUMO tag, the expression level of the fusion protein significantly increased, and it also improved the solubility of the recombinant protein.

### 3.2 Optimization of SUMO-EL1 and SUMO-RE1 expression

**FIGURE 1:**
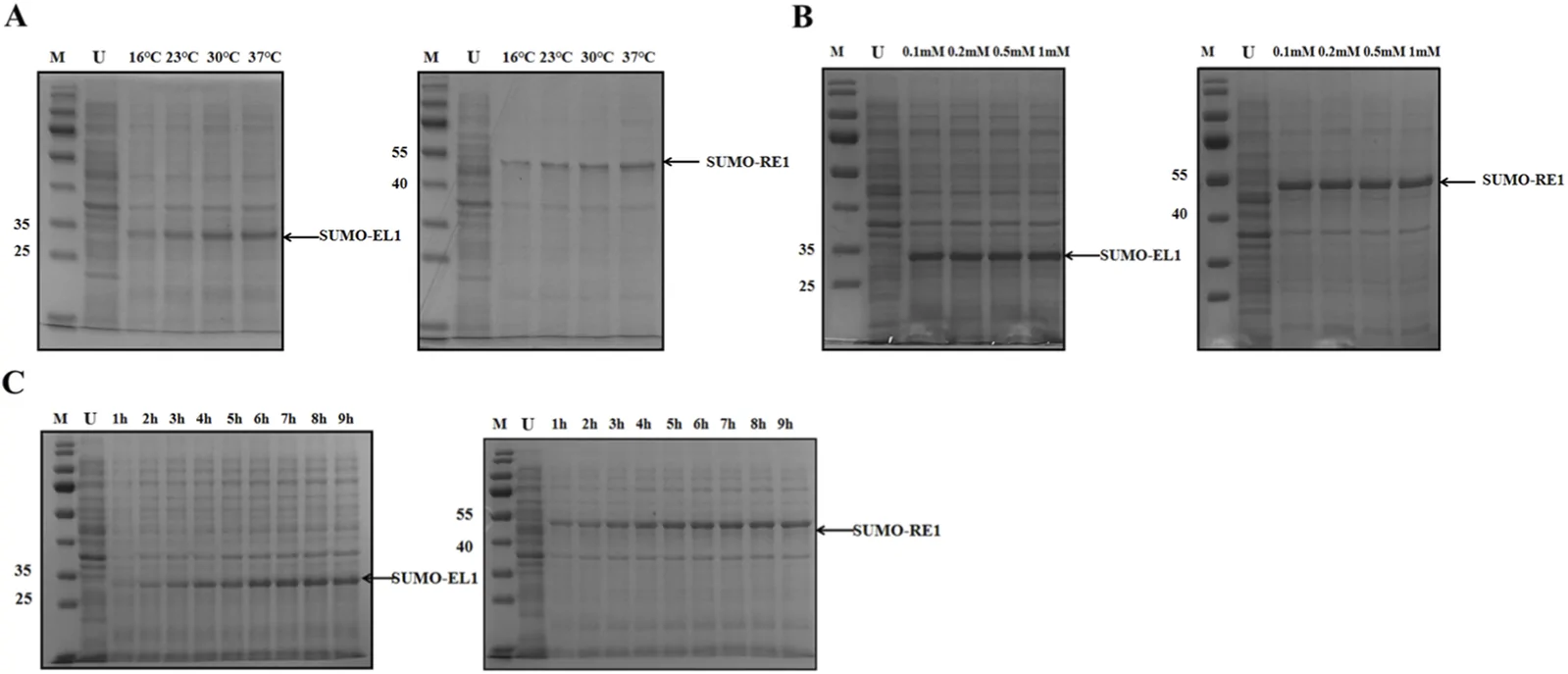
Optimization of SUMO-EL1 and SUMO-RE1 expression in a 1 L shake flask. The expression of SUMO-EL1 and SUMO-RE1 at different culture conditions, including expression temperature **(A)** IPTG concentration **(B)** and expression time after IPTG induction **(C)**. The supernatant of cell lysate at different culture conditions were analyzed by SDS-PAGE. For expression temperature, lanes from left to right, cell lysate supernatant after induction at 16, 23, 30 °C and 37 °C. For IPTG concentration, lanes from left to right, cell lysate supernatant after induction at 0.1, 0.2, 0.5 and 1 mM IPTG. For expression time, lanes from left to right, cell lysate supernatant at 1, 2, 3, 4, 5, 6, 7, 8 and 9 h after induction. M: Marker; U: non-enzymolysis.

To determine the optimal conditions for shake-flask fermentation of the engineered strains, a single-factor experimental approach was employed. The optimized shake-flask fermentation conditions included expression temperature, IPTG concentration, and IPTG induction time. The results showed that protein expression levels were highest when induced at 37 °C (Figure 1A). When the IPTG concentration ranged from 0.1 mM to 1 mM, there was no significant change in the expression levels of the soluble fusion proteins SUMO-EL1 and SUMO-RE1 (Figure 1B). Considering the industrial costs for subsequent scale-up fermentation, the lowest concentration of 0.1 mM was selected as the optimal induction concentration. As shown in Figure 1C, the expression levels of both proteins remained relatively unchanged after 7 h of IPTG induction, so 7 h was chosen as the induction time for the culture conditions.

### 3.3 Purification of EL1 and RE1

**FIGURE 2:**
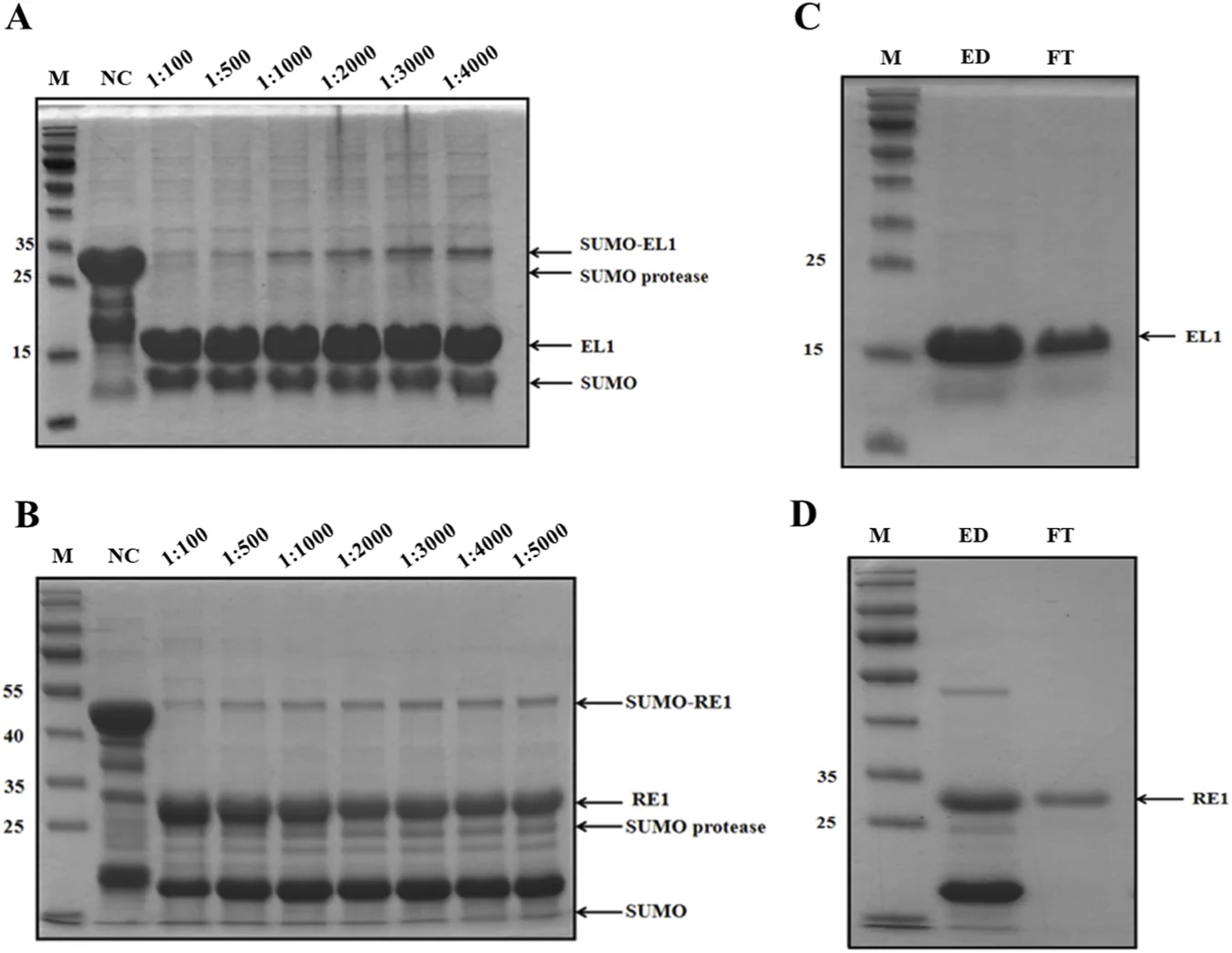
Production of the recombinant EL1 and RE1 protein. **(A)** The optimal mass ratio of fusion protein SUMO-EL1 digestion. **(B)** The optimal mass ratio of fusion protein SUMO-RE1 digestion. **(C)** Purification of EL1. **(D)** Purification of RE1. M: Marker; NC: Negative Control, undigested fusion protein; ED: enzyme digestion; FT: flow-through.

The SUMO-EL1 and SUMO-RE1 were eluted in a 250 mM imidazole solution. The results, as shown in Supplementary Figure S2, indicate that the first step of purification successfully removed a portion of the impurity protein from both fusion proteins. As shown in Figures 2A,B, the enzymatic hydrolysis reaction for SUMO-RE1 and SUMO-EL1 was performed with a mass ratio (enzyme∶ protein) of 1: 100 at 4 °C for 2 h. Since the protein contain the 6×His tag, the SUMO tag, SUMO-EL1 and SUMO-RE1 were loaded onto an Ni-NTA affinity chromatography column, while EL1 and RE1 were collected in the flow-through. The target protein was separated from other protein. The purified proteins were quantified using a BCA protein assay kit, and their purity was analyzed by SDS-PAGE and Coomassie Brilliant Blue staining (Figures 2C,D). Finally, grayscale scanning analysis revealed that the purity of EL1 and RE1 was 98.17% and 99.38%, respectively, indicating the successful purification of the target proteins EL1 and RE1.

### 3.4 Bioactivity *in vitro* of EL1 and RE1

**FIGURE 3:**
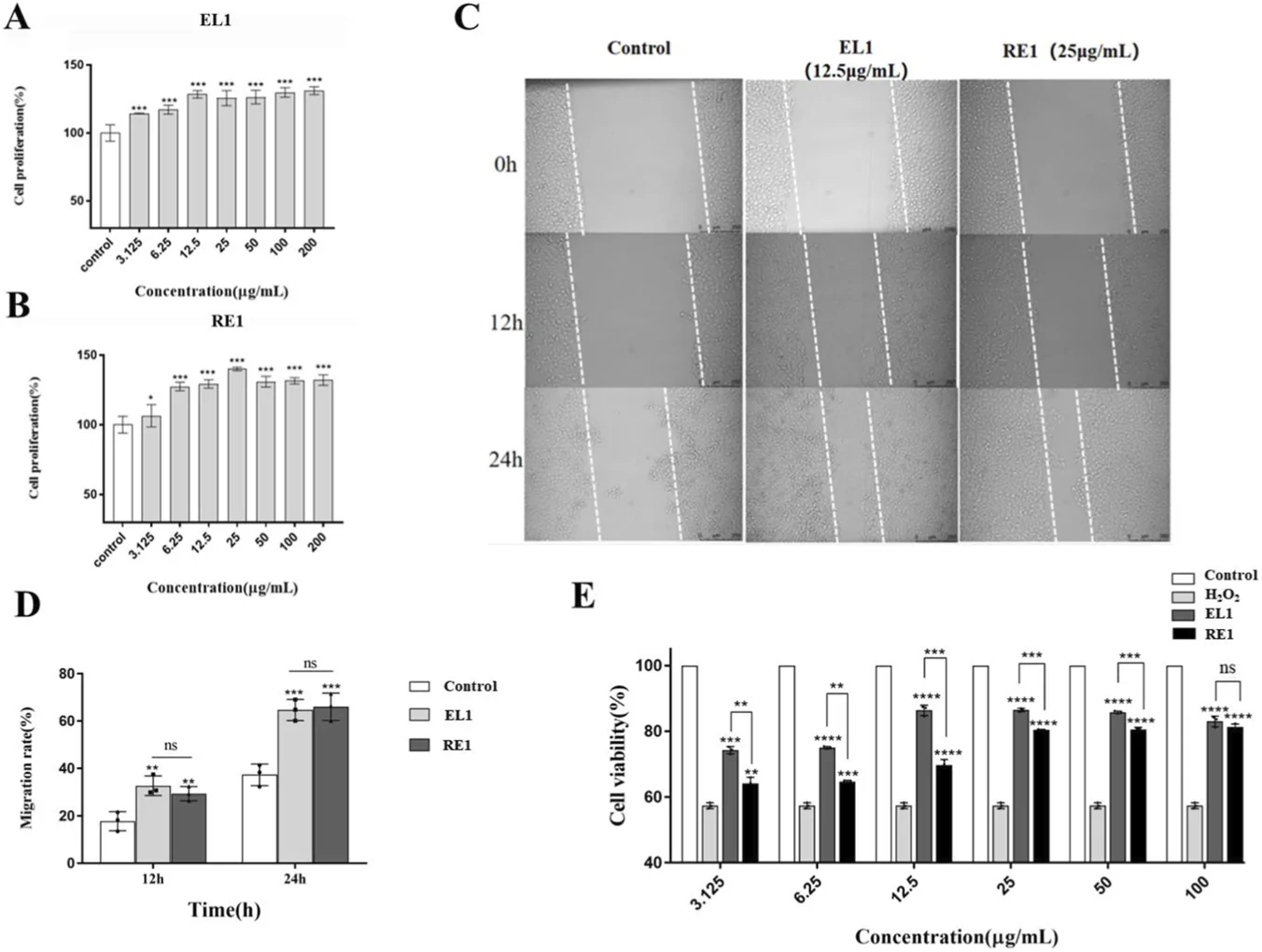
Bioactivity of EL1 and RE1 *in vitro*. Cell viability of 3T3 cells were treated with EL1 **(A)** and RE1 **(B)** for 24 h **(C)** 3T3 cell migration ability evaluated by scratch assay. **(D)** Statistical analysis of 3T3 cells scratch assay. **(E)** Antioxidant activity assay of EL1 and RE1 treated by H_2_O_2_. The data are expressed as mean ± standard deviation (SD), *p < 0.05. **p < 0.01. ***p < 0.001 and ****p < 0.0001.

The effects of two elastins, EL1 and RE1, on the proliferation of 3T3 cells at different concentrations were investigated using the CCK-8 assay, and the results were compared (Figures 3A,B). According to the cell proliferation assay results, compared to the blank control group, both EL1 and RE1 promoted the proliferation of 3T3 cells within a certain concentration range, and their proliferative effects exhibited a dose-dependent relationship. For EL1, doses of 12.5, 100, and 200 μg/mL all demonstrated good proliferative promoting effects, taking into account the principle of the ‘minimum effective dose’ of the drug, we chose 12.5 μg/mL for subsequent functional analysis, thereby minimizing costs and reducing potential toxicity risks. The maximum proliferation rate of 35.38% was achieved for RE1 at a concentration of 25 μg/mL. Therefore, in subsequent experiments, EL1 was used at a concentration of 12.5 μg/mL, and RE1 was used at a concentration of 25 μg/mL.

In the 3T3 cell migration experiment, the results are shown in Figures 3C,D. The migration rates for the Control, EL1 (12.5 μg/mL), and RE1 (25 μg/mL) groups at 12 h were 17% ± 6.25%, 32.72% ± 10.76%, and 29.82% ± 9.09%, respectively. At 24 h, the rates were 37.38% ± 11.50%, 64.69% ± 9.90%, and 66.04% ± 7.32%, respectively. Compared to the Control group, both proteins significantly promoted wound healing at 12 and 24 h, with statistical differences (12 h, p < 0.01, 24 h, p < 0.001).

ELN is known to possess antioxidant and anti-aging properties (Sun et al., 2023; Bergonzi et al., 2020). To explore whether EL1 and RE1 exhibit antioxidant capabilities in 3T3 cells, we employed a hydrogen peroxide (H_2_O_2_)-induced damage model. We selected 3 mM H_2_O_2_ to treat 3T3 cells for 24 h (Supplementary Figure S3), resulting in a cell viability of approximately 53%, which served as the basis for subsequent experiments. As shown in Figure 3E, we observed that both EL1 and RE1 at different concentrations exhibited protective effects against oxidative stress, with statistically significant differences between treatments when the protein concentrations were below 25 μg/mL. At an EL1 concentration of 12.5 μg/mL, the optimal protective effect against oxidative damage was achieved, with cell viability recovering to 84.34% ± 3.57% compared to the H_2_O_2_-treated group (p < 0.0001). The optimal protective concentration of RE1 was 25 μg/mL, with cell viability recovering to 80.67% ± 2.08% (p < 0.0001).

### 3.5 EL1 and RE1 promote dermal regeneration and collagen deposition

**FIGURE 4:**
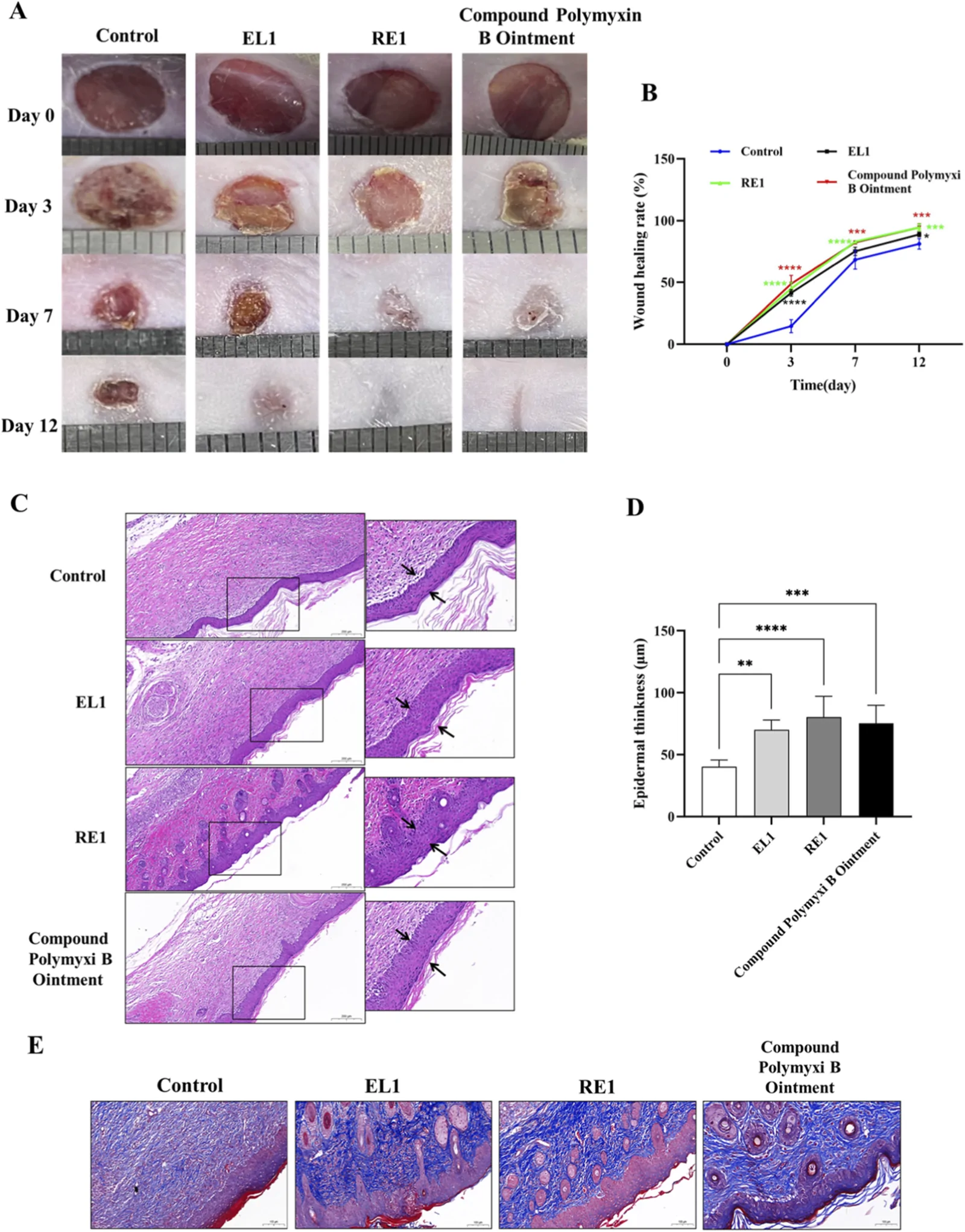
Effects of EL1 and RE1 on wound closure in mice skin and analysis of wounds morphology. **(A)** Representative images of the wounds on the 0th, 3rd, 7th and 12th day. **(B)** Statistical analysis of the wound closure rate. **(C)** Representative images of H&E staining images of skin tissue on day 7 (n = 3). Scale bar = 200 μm. **(D)** Statistical analysis of epidermal thickness based on the H&E staining. **(E)** Representative images of Masson’s trichrome staining of skin tissue on day 7 (n = 3). Red indicated red blood cells, muscle fibers, or cytoplasm, and blue indicated for collagen or fibers. Scale bar = 100 μm. The data are expressed as mean ± SD, *p < 0.05. **p < 0.01. ***p < 0.001 and ****p < 0.0001.

Next, we investigated the effects of these two proteins on full-thickness wound healing in mice. As shown in Figure 4A, compared with the control group, the wounds in the drug treatment group were almost completely healed on the 12th day. In Figure 4B, compared to the control group, the RE1 group showed a trend of accelerated wound healing during the first 3 days better than EL1. After the third day, the wound healing rate significantly accelerated in both the RE1 and EL1 treatment groups. Specifically, the RE1 group reached a wound healing rate of 83.12% ± 1.13% on the 7th day, which was slightly higher than that of the compound polymyxin B ointment group (82.43% ± 1.26%), and was significantly better than that of the control group (68.38% ± 7.57%, p < 0.0001). However, the recovery rate in the EL1 group (75.26% ± 3.31%) was slower than that in the RE1 group. On the 12th day, the wounds in all treatment groups, except the control group, had almost completely healed, and the wounds in the RE1 group healed more smoothly and with minimal scarring.

We analyzed the regenerated skin by observing and quantifying the epidermal thickness of the mice on the seventh day (Figure 4C). As shown in Figure 4D, the epidermal thickness in the elastin EL1, RE1, and compound polymyxin B ointment groups showed statistically significant differences compared to the untreated group (p < 0.01), and the RE1 treatment group exhibited the best skin recovery at the wound site. Therefore, although compound polymyxin B ointment accelerated wound healing during the first 3 days, treatment with elastin RE1 promoted re-epithelialization of the wound and facilitated the restoration of normal epidermal structure in mice. Additionally, collagen, as a major component of the skin extracellular matrix, plays a crucial role in the synthesis and deposition during wound healing and repair (Halper and Kjaer, 2014). Therefore, we further evaluated wound recovery using Masson staining, as shown in Figure 4E. Except for the Control group, where a small amount of scabbing was still observed, a large amount of new blue collagen fibers was observed in all other treatment groups. This may explain the significant reduction in scar formation in the regenerated skin of mice treated with elastin.

### 3.6 EL1 and RE1 promote the infiltration of macrophages

**FIGURE 5:**
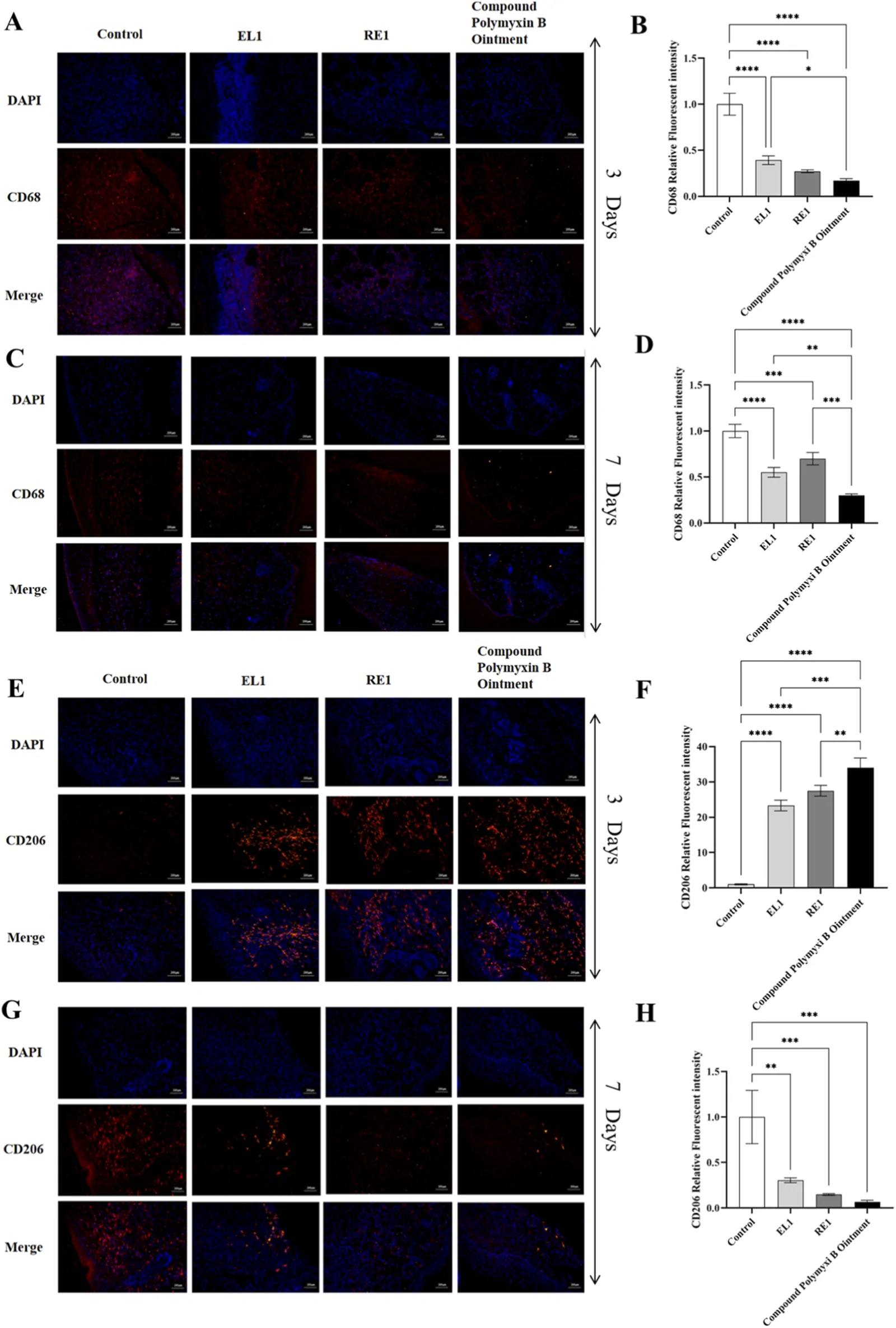
Effects of EL1 and RE1 on the infiltration of macrophages. **(A)** Representative images of CD68 immunofluorescence staining of wound tissues on day 3. Scale bar = 200 μm. **(B)** CD68 relative fluorescence intensity on day 3 (n = 3). **(C)** Representative images of CD68 immunofluorescence staining of wound tissues on day 7. Scale bar = 200 μm. **(D)** CD68 relative fluorescence intensity on day 7 (n = 3). **(E)** Representative images of CD206 immunofluorescence staining of wound tissues on day 3. Scale bar = 200 μm. **(F)** CD206 relative fluorescence intensity on day 3 (n = 3). **(G)** Representative images of CD206 immunofluorescence staining of wound tissues on day 7. Scale bar = 200 μm. **(H)** CD206 relative fluorescence intensity on day 7 (n = 3). (*p < 0.05. **p < 0.01. ***p < 0.001 and ****p < 0.0001).

CD68 is a marker for M1-type macrophages during the inflammatory phase, and CD206 is a marker for M2-type macrophages (Hsu et al., 2023; Choudhary and Malek, 2023). We conducted immunofluorescence staining for CD68 and CD206 to preliminarily assess the mechanism by which elastin promotes wound healing. As shown in Figure 5, by comparing the immunofluorescence of CD68 in mouse wounds on the third and seventh days (Figures 5A–D), we observed that on the 3rd day, all groups exhibited a certain degree of inflammatory response. And compared with the control group, the inflammatory response at the wounds in the treatment groups were significantly reduced. On the 7th day, the control group still displayed some inflammatory response, whereas the inflammatory response in all treatment groups had largely subsided. These results indicate that EL1 andRE1 mitigated the inflammatory response during the first 3 days and promoted wound healing, which aligns with the macroscopic wound recovery results observed earlier.

Next, we further validated the effects of each treatment group on macrophages through the immunofluorescence results of CD206. As shown in Figures 5E,F, on the 3rd day, the CD206 fluorescence response in all treatment groups was stronger than that in the Control group, with statistically significant differences. Combined with the CD68 immunofluorescence results in Figure 5A, this indicates that after treatment with EL1 and RE1, more M1-type macrophages transitioned to M2-type, which is consistent with the inflammatory response observed on the third day. By the 7th day, the Control group exhibited the strongest CD206 fluorescence response, while the expression levels of CD206 in the other treatment groups stabilized and became consistent (Figures 5G,H). This suggests that the inflammatory response in the other groups had largely subsided by the seventh day, whereas the Control group still maintained a higher level of anti-inflammatory activity. This finding aligns with the results of wound healing area and CD68 expression levels observed on the 7th day.

### 3.7 EL1 and RE1 promote the formation of angiogenesis

**FIGURE 6:**
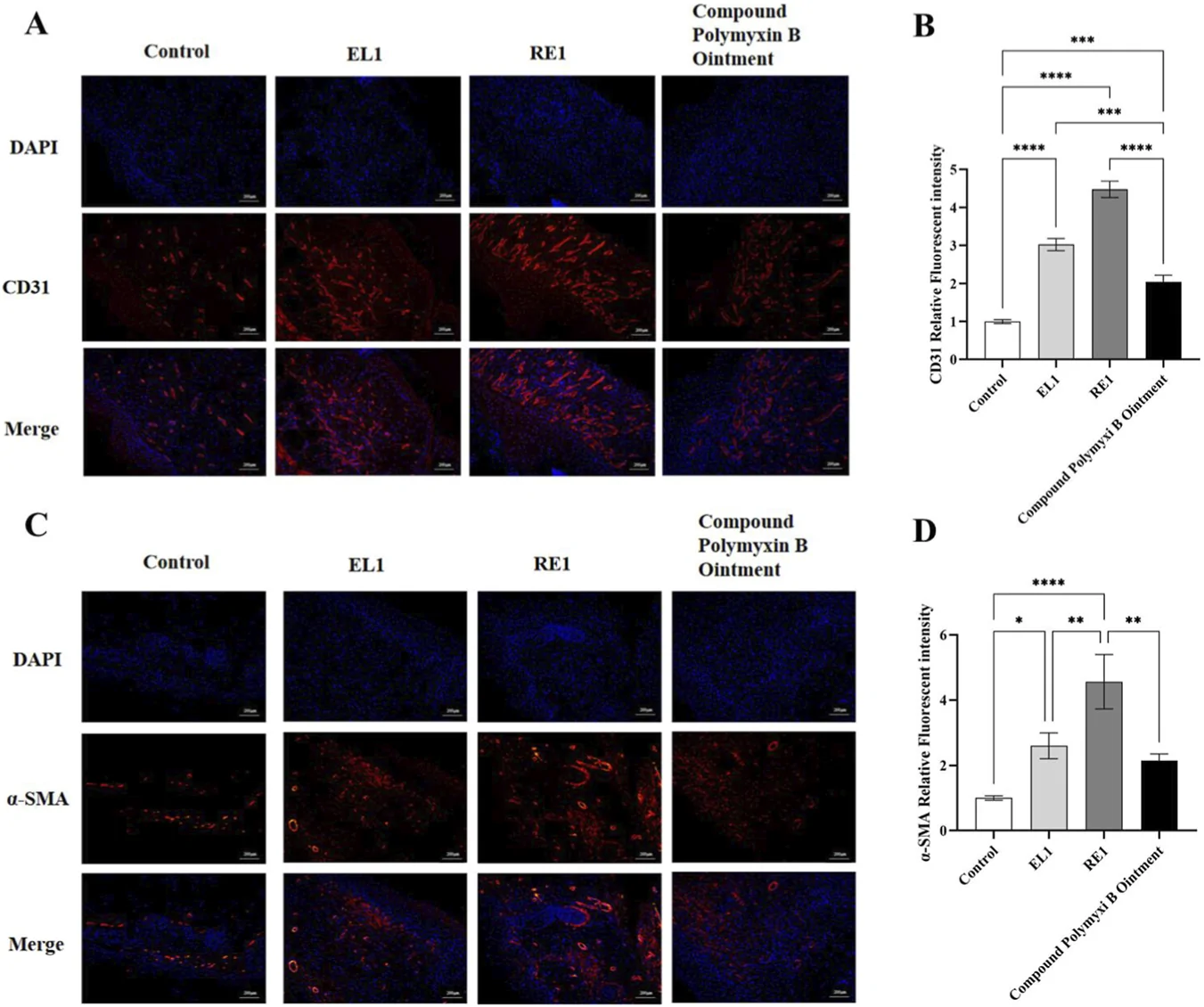
Effects of EL1 and RE1 on angiogenesis. **(A)** Immunofluorescence staining of CD31 (red) of wound tissues on day 7 (scale bars: 200 μm). **(B)** CD31 relative fluorescence intensity (n = 3). **(C)** Immunofluorescence staining of α-SMA (red) of wound tissues on day 7 (scale bars: 200 μm). **(D)** α-SMA relative fluorescence intensity (n = 3). (*p < 0.05. **p < 0.01. ***p < 0.001 and ****p < 0.0001).

Wound healing is closely associated with the formation of new blood vessels. CD31, also known as platelet endothelial cell adhesion molecule, is a marker of endothelial differentiation and plays a role in angiogenesis. The immunofluorescence results of CD31 in mouse wounds on the seventh day (Figures 6A,B) revealed that after treatment with EL1 and RE1, the expression of CD31 in the newly formed tissue significantly increased and was superior to that in the compound polymyxin B ointment group. This suggests that the two recombinant elastins may accelerate wound healing by promoting angiogenesis.

α-SMA, a marker for smooth muscle cells, is currently recognized as an indicator of myofibroblasts (Younesi et al., 2021). By detecting the expression of α-SMA, we can further determine the effects of different treatment groups on neovascularization in mouse wounds. According to the results in Figures 6C,D, we found that after treatment with EL1 and RE1, the expression of α-SMA in the newly formed tissue of mice was significantly higher than that in the control group and better than that in the compound polymyxin B ointment group. This is consistent with the aforementioned CD31 results, further confirming that treatment with our recombinant elastins promotes the formation of new blood vessels in mouse wounds.

## 4 Discussion

In recent years, biomaterials based on elastin have gained increasing attention in fields such as wound repair dressings and drug delivery (Chambre et al., 2020; Santos et al., 2019; Ahmad Hariza et al., 2023). Currently, the primary method of obtaining elastin is through extraction from animal tissues, which presents issues such as immunogenicity and poor water solubility. Achieving large-scale production of recombinant proteins through synthetic biology has become a popular research topic in recent years. For a long time, the use of the *Escherichia coli* system to produce recombinant elastin, especially full-length or complex human sequences, has been limited by issues such as poor solubility, instability, and the formation of inclusion bodies (Reichheld et al., 2014). Although various fusion tags (such as GST, MBP) have been attempted to promote solubility, most of them are only effective for simple repetitive peptides, and the tag removal steps often result in yield loss or the introduction of unnatural terminus (Zhu et al., 2025; Chen et al., 2015; Martin et al., 1995; Bataille et al., 2015). In this work, we have made significant improvements over existing technologies by introducing a SUMO fusion strategy combined with a streamlined two-step purification scheme. EL1, the structure is mainly composed of a repeating unit of five amino acids (VPGXG), which are linked together in series, and the amino acid X represents any amino acid other than proline. RE1 is entirely based on human elastin with high biomimicry. It is notable that this method not only significantly enhances the soluble expression of conventional ELP (EL1), but more importantly, achieves the soluble expression of a human elastin sequence (RE1) with excellent biological activity that is extremely difficult to express on its own-transforming its expression state from ‘undetectable’ to a high purified product. This process eliminates the potential interference of labels on the activity, providing a reliable protein source for subsequent activity and material applications. The successful preparation of these two recombinant elastins provides a foundation for the high-throughput production of full-length or complex human elastin using prokaryotic expression systems. Given the promising performance at this laboratory scale, we anticipate that the yield could be further enhanced through fed-batch fermentation or bioreactor scale-up in future studies.

To determine whether the two novel recombinant proteins, EL1 and RE1, designed and expressed in this study possess biological activity, we tested their activities *in vitro*, including their ability to promote 3T3 cell proliferation, migration and antioxidant capacity, as well as their effectiveness *in vivo* in promoting wound healing in mice. From our research, it is evident that in the cell scratch assay, both EL1 and RE1 significantly promoted cell migration at 12 and 24 h. In the antioxidant assay, EL1 and RE1 were able to increase the viability of cells after oxidative damage.

Since EL1 and RE1 demonstrated good activity *in vitro*, we were interested in whether EL1 and RE1 might contribute to wound repair *in vivo*. As shown in previous studies, ELPs and tropoelastin have a positive effect on wound healing (Koria et al., 2011; Wang et al., 2024). Therefore, we further explored the role of the designed EL1 and RE1 in wound healing through animal experiments. The experimental results demonstrated that both designed recombinant elastins, EL1 and RE1, could accelerate wound healing. Although the healing rate was slightly slower compared to commonly used wound medications, this is likely due to the limited efficacy of using elastin alone. However, we observed that the wounds treated with our designed elastins healed with smoother skin and reduced scar formation, suggesting that our designed elastins has potential applications in biomaterials. Based on *in vitro* experiments, our designed elastins promoted the proliferation and migration of 3T3 mouse fibroblasts, which may play a significant role in dermal remodeling, as evidenced by the HE staining results of mouse wound skin. Additionally, Masson staining results showed that a large amount of new blue collagen fibers was observed in treatment groups.

Wound healing generally consists of four stages: hemostasis, inflammation, proliferation, and maturation (Eming et al., 2014). To further elucidate the mechanism by which the recombinant elastin promotes wound healing, we conducted immunofluorescence assays to detect relevant factors. Macrophages play a significant role in different stages of wound healing (Vannella and Wynn, 2017). For example, M1 (or M1-like) macrophages produce pro-inflammatory factors to control infection during the early inflammatory phase, while M2 (or M2-like) macrophages produce anti-inflammatory factors to resolve inflammation (Yunna et al., 2020). Furthermore, M2 (or M2-like) macrophages secrete growth factors that promote re-epithelialization and angiogenesis, facilitating wound healing. Based on the immunofluorescence results of CD68/CD206, the recombinant human elastins EL1 and RE1 could rapidly increase the expression of CD206, and regulate the function of macrophages *in vivo*, shifting from a pro-inflammatory to an anti-inflammatory/pro-healing phenotype. Additionally, neovascularization and angiogenesis are critical elements in the outcome of wound healing (Bodnar, 2015). Newly formed blood vessels participate in the healing process by providing nutrients and oxygen, as well as creating pathways for cells to enter the growing tissue. High expression of the cell adhesion molecule CD31 promotes blood vessel formation and wound healing (Hache et al., 2016). Overall, the results show that RE1 exhibits superior biological activity compared to EL1, which is likely related to its sequence. When comparing the sequences of EL1 and RE1, RE1, with its more abundant functional domains such as cell adhesion and signal transduction, can more effectively participate in and regulate cell behavior, thus demonstrating a better overall effect. The repeating unit VPGXG of EL1 does not contain a complete active sequence. Although it has conformational changes, it lacks a high-order structure, and thus its specific interaction with cell surface receptors (such as elastin receptor complexes) is weakened, resulting in weaker cell regulatory functions than RE1. Our study displayed that the recombinant human elastins EL1 and RE1 increased the expression of CD31 and α-SMA to promote angiogenesis, which indicating that the recombinant human elastin can create a favorable regenerative microenvironment driving skin cells in wound to promote tissue remodeling.

## 5 Conclusion

In summary, we provided a new method for recombinant elastin synthesis to solve the problems of single sequence, low expression and low bionic degree of elastin prokaryotic expression system, and successfully designed two new recombinant human elastins with good biological activity and can promote wound healing in mice, which may have promising clinical applications in skin wound repair. In the follow-up study, the physical and chemical properties of the two elastins can be further studied to determine whether they can be applied to biological and medical materials. In addition, biological activity and mechanism of action in the animal model can be further verified.

## Data Availability

The original contributions presented in the study are included in the article/Supplementary Material, further inquiries can be directed to the corresponding author.

## References

[B1] Ahmad Hariza A. M. Mohd Yunus M. H. Fauzi M. B. Murthy J. K. Tabata Y. Hiraoka Y. (2023). The fabrication of gelatin-elastin-nanocellulose composite bioscaffold as a potential acellular skin substitute. Polym. (Basel) 15 (3), 779. 10.3390/polym15030779 36772084 PMC9920652

[B2] Bataille L. Dieryck W. Hocquellet A. Cabanne C. Bathany K. Lecommandoux S. (2015). Expression and purification of short hydrophobic elastin-like polypeptides with maltose-binding protein as a solubility tag. Protein Expr. Purif. 110, 165–171. 10.1016/j.pep.2015.03.013 25819942

[B3] Bataille L. Dieryck W. Hocquellet A. Cabanne C. Bathany K. Lecommandoux S. (2016). Recombinant production and purification of short hydrophobic elastin-like polypeptides with low transition temperatures. Protein Expr. Purif. 121, 81–87. 10.1016/j.pep.2016.01.010 26802681

[B4] Baumann L. Bernstein E. F. Weiss A. S. Bates D. Humphrey S. Silberberg M. (2021). Clinical relevance of elastin in the structure and function of skin. Aesthet. Surg. J. Open Forum 3 (3), ojab019. 10.1093/asjof/ojab019 34195612 PMC8239663

[B5] Bergonzi C. d'Ayala G. G. Elviri L. Laurienzo P. Bandiera A. Catanzano O. (2020). Alginate/human elastin-like polypeptide composite films with antioxidant properties for potential wound healing application. Int. J. Biol. Macromol. 164, 586–596. 10.1016/j.ijbiomac.2020.07.084 32679321

[B6] Bodnar R. J. (2015). Chemokine regulation of angiogenesis during wound healing. Adv. Wound Care (New Rochelle) 4 (11), 641–650. 10.1089/wound.2014.0594 26543678 PMC4620517

[B7] Carlos Rodríguez-Cabello J. Reguera J. Girotti A. Alonso M. Testera A. M. (2005). Developing functionality in elastin-like polymers by increasing their molecular complexity: the power of the genetic engineering approach. Prog. Polym. Sci. 30 (11), 1119–1145. 10.1016/j.progpolymsci.2005.07.004

[B8] Chambre L. Martín-Moldes Z. Parker R. N. Kaplan D. L. (2020). Bioengineered Elastin- and silk-biomaterials for drug and gene delivery. Adv. Drug Deliv. Rev. 160, 186–198. 10.1016/j.addr.2020.10.008 33080258 PMC7736173

[B9] Chen X. Shi J. Chen R. Wen Y. Shi Y. Zhu Z. (2015). Molecular chaperones (Trxa, sumo, intein, and gst) mediating expression, purification, and antimicrobial activity assays of plectasin in Escherichia coli. Biotechnol. Appl. Biochem. 62 (5), 606–614. 10.1002/bab.1303 25311837

[B10] Choudhary M. Malek G. (2023). Cd68: potential contributor to inflammation and Rpe cell dystrophy. Adv. Exp. Med. Biol. 1415, 207–213. 10.1007/978-3-031-27681-1_30 37440035

[B11] de Oliveira S. Rosowski E. E. Huttenlocher A. (2016). Neutrophil migration in infection and wound repair: going forward in reverse. Nat. Rev. Immunol. 16 (6), 378–391. 10.1038/nri.2016.49 27231052 PMC5367630

[B12] Depenveiller C. Baud S. Belloy N. Bochicchio B. Dandurand J. Dauchez M. (2024). Structural and physical basis for the elasticity of elastin. Q. Rev. Biophys. 57, e3. 10.1017/S0033583524000040 38501287

[B13] Eming S. A. Paul M. Tomic-Canic M. (2014). Wound repair and regeneration: mechanisms, signaling, and translation. Sci. Transl. Med. 6 (265), 265sr6. 10.1126/scitranslmed.3009337 25473038 PMC4973620

[B14] Frech S. Lichtenberger B. M. (2024). Modulating embryonic signaling pathways paves the way for regeneration in wound healing. Front. Physiol. 15, 1367425. 10.3389/fphys.2024.1367425 38434140 PMC10904466

[B15] Gardeazabal L. Izeta A. (2024). Elastin and collagen fibres in cutaneous wound healing. Exp. Dermatol 33 (3), e15052. 10.1111/exd.15052 38483134

[B16] Hache G. Garrigue P. Bennis Y. Stalin J. Moyon A. Cerami A. (2016). Ara290, a specific agonist of Erythropoietin/Cd131 heteroreceptor, improves circulating endothelial progenitors' angiogenic potential and homing ability. Shock 46 (4), 390–397. 10.1097/SHK.0000000000000606 27172159

[B17] Halper J. Kjaer M. (2014). Basic components of connective tissues and extracellular matrix: elastin, fibrillin, fibulins, fibrinogen, fibronectin, laminin, tenascins and thrombospondins. Adv. Exp. Med. Biol. 802, 31–47. 10.1007/978-94-007-7893-1_3 24443019

[B18] Hedtke T. Schräder C. U. Heinz A. Hoehenwarter W. Brinckmann J. Groth T. (2019). A comprehensive map of human elastin cross-linking during elastogenesis. Febs J. 286 (18), 3594–3610. 10.1111/febs.14929 31102572

[B19] Hsu C. H. Pan Y. J. Zheng Y. T. Lo R. Y. Yang F. Y. (2023). Ultrasound reduces inflammation by modulating M1/M2 polarization of microglia through Stat1/Stat6/Pparγ signaling pathways. CNS Neurosci. Ther. 29 (12), 4113–4123. 10.1111/cns.14333 37401041 PMC10651950

[B20] Isenburg J. C. Karamchandani N. V. Simionescu D. T. Vyavahare N. R. (2006). Structural requirements for stabilization of vascular elastin by polyphenolic tannins. Biomaterials 27 (19), 3645–3651. 10.1016/j.biomaterials.2006.02.016 16527345

[B21] Kakinoki S. Yamaoka T. (2014). Thermoresponsive elastin/laminin mimicking artificial protein for modifying plla scaffolds in nerve regeneration. J. Mater Chem. B 2 (31), 5061–5067. 10.1039/c4tb00305e 32261839

[B22] Koria P. Yagi H. Kitagawa Y. Megeed Z. Nahmias Y. Sheridan R. (2011). Self-assembling elastin-like peptides growth factor chimeric nanoparticles for the treatment of chronic wounds. Proc. Natl. Acad. Sci. U. S. A. 108 (3), 1034–1039. 10.1073/pnas.1009881108 21193639 PMC3024670

[B23] Li J. Huang W. He H. Shi S. Sun X. Xiao J. (2023). Biocompatible and bioactive hydrogels of recombinant fusion elastin with low transition temperature for improved healing of Uv-Irradiated skin. J. Mater Chem. B 11 (29), 6975–6982. 10.1039/d3tb00564j 37401183

[B24] Li D. Wang Y. Zhu S. Hu X. Liang R. (2024). Recombinant fibrous protein biomaterials meet skin tissue engineering. Front. Bioeng. Biotechnol. 12, 1411550. 10.3389/fbioe.2024.1411550 39205856 PMC11349559

[B25] Martin S. L. Vrhovski B. Weiss A. S. (1995). Total synthesis and expression in Escherichia coli of a gene encoding human tropoelastin. Gene 154 (2), 159–166. 10.1016/0378-1119(94)00848-m 7890158

[B26] Mithieux S. M. Wise S. G. Weiss A. S. (2013). Tropoelastin--a multifaceted naturally smart material. Adv. Drug Deliv. Rev. 65 (4), 421–428. 10.1016/j.addr.2012.06.009 22784558

[B27] Mora H. Angela C. Schmelzer C. E. H. Hoehenwarter W. Heyroth F. Heinz A. (2016). Molecular-level insights into aging processes of skin elastin. Biochimie 128-129, 163–173. 27569260 10.1016/j.biochi.2016.08.010

[B28] Ozsvar J. Yang C. Cain S. A. Baldock C. Tarakanova A. Weiss A. S. (2021). Tropoelastin and elastin assembly. Front. Bioeng. Biotechnol. 9, 643110. 10.3389/fbioe.2021.643110 33718344 PMC7947355

[B29] Peña O. A. Martin P. (2024). Cellular and molecular mechanisms of skin wound healing. Nat. Rev. Mol. Cell Biol. 25 (8), 599–616. 10.1038/s41580-024-00715-1 38528155

[B30] Rawat K. Shrivastava A. (2022). Neutrophils as emerging protagonists and targets in chronic inflammatory diseases. Inflamm. Res. 71 (12), 1477–1488. 10.1007/s00011-022-01627-6 36289077 PMC9607713

[B31] Reichheld S. E. Muiznieks L. D. Stahl R. Simonetti K. Sharpe S. Keeley F. W. (2014). Conformational transitions of the cross-linking domains of elastin during self-assembly. J. Biol. Chem. 289 (14), 10057–10068. 10.1074/jbc.M113.533893 24550393 PMC3974977

[B32] Rodgers U. R. Weiss A. S. (2005). Cellular interactions with elastin. Pathol. Biol. 53 (7), 390–398. 10.1016/j.patbio.2004.12.022 16085115

[B33] Rodrigues M. Kosaric N. Bonham C. A. Gurtner G. C. (2019). Wound healing: a cellular perspective. Physiol. Rev. 99 (1), 665–706. 10.1152/physrev.00067.2017 30475656 PMC6442927

[B34] Sakai L. Y. Keene D. R. Engvall E. (1986). Fibrillin, a new 350-Kd glycoprotein, is a component of extracellular microfibrils. J. Cell Biol. 103 (6), 2499–2509. 10.1083/jcb.103.6.2499 3536967 PMC2114568

[B35] Santos M. Serrano-Dúcar S. González-Valdivieso J. Vallejo R. Girotti A. Cuadrado P. (2019). Genetically engineered elastin-based biomaterials for biomedical applications. Curr. Med. Chem. 26 (40), 7117–7146. 10.2174/0929867325666180508094637 29737250

[B36] Shapiro S. D. Endicott S. K. Province M. A. Pierce J. A. Campbell E. J. (1991). Marked longevity of human lung parenchymal elastic fibers deduced from prevalence of D-Aspartate and nuclear weapons-related radiocarbon. J. Clin. Invest 87 (5), 1828–1834. 10.1172/JCI115204 2022748 PMC295305

[B37] Sun Q. Zhang Xu Gao M. Zhang C. Peng B. (2023). Resource utilization of bovine neck ligament: enzymatic preparation of elastin peptide and its antioxidant activity. Appl. Biochem. Biotechnol. 195 (1), 33–50. 10.1007/s12010-022-04102-4 35932368

[B38] Uitto J. Christiano A. M. Kähäri V.-M. Bashir M. M. Joel R. (1991). Molecular biology and pathology of human elastin. Biochem. Soc. Trans. 19 (4), 824–829. 10.1042/bst0190824 1794566

[B39] Vannella K. M. Wynn T. A. (2017). Mechanisms of organ injury and repair by macrophages. Annu. Rev. Physiol. 79, 593–617. 10.1146/annurev-physiol-022516-034356 27959618

[B40] Veith A. P. Henderson K. Spencer A. Sligar A. D. Baker A. B. (2019). Therapeutic strategies for enhancing angiogenesis in wound healing. Adv. Drug Deliv. Rev. 146, 97–125. 10.1016/j.addr.2018.09.010 30267742 PMC6435442

[B41] Vindin H. Mithieux S. M. Weiss A. S. (2019). Elastin architecture. Matrix Biol. 84, 4–16. 10.1016/j.matbio.2019.07.005 31301399

[B42] Vrhovski B. Weiss A. S. (1998). Biochemistry of tropoelastin. Eur. J. Biochem. 258 (1), 1–18. 10.1046/j.1432-1327.1998.2580001.x 9851686

[B43] Wang Z. Shi H. Silveira P. A. Mithieux S. M. Wong W. C. Liu L. (2024). Tropoelastin modulates systemic and local tissue responses to enhance wound healing. Acta Biomater. 184, 54–67. 10.1016/j.actbio.2024.06.009 38871204

[B44] Weihermann A. C. Lorencini M. Brohem C. A. de Carvalho C. M. (2017). Elastin structure and its involvement in skin photoageing. Int. J. Cosmet. Sci. 39 (3), 241–247. 10.1111/ics.12372 27731897

[B45] Wen Q. Mithieux S. M. Weiss A. S. (2020). Elastin biomaterials in dermal repair. Trends Biotechnol. 38 (3), 280–291. 10.1016/j.tibtech.2019.08.005 31870589

[B46] Yao X. L. Conticello V. P. Hong M. (2004). Investigation of the dynamics of an elastin-mimetic polypeptide using solid-state nmr. Magn. Reson Chem. 42 (2), 267–275. 10.1002/mrc.1330 14745807

[B47] Younesi F. S. Son D. O. Firmino J. Hinz B. (2021). Myofibroblast markers and microscopy detection methods in cell culture and histology. Methods Mol. Biol. 2299, 17–47. 10.1007/978-1-0716-1382-5_3 34028733

[B48] Yunna C. Mengru H. Lei W. Weidong C. (2020). Macrophage M1/M2 polarization. Eur. J. Pharmacol. 877, 173090. 10.1016/j.ejphar.2020.173090 32234529

[B49] Zhu S. Yu Y. Ren Y. Xu L. Wang H. Ling X. (2021). The emerging roles of neutrophil extracellular traps in wound healing. Cell Death Dis. 12 (11), 984. 10.1038/s41419-021-04294-3 34686654 PMC8536667

[B50] Zhu L. Shi J. Li F. Wang X. Sun X. Xiao J. (2025). Recombinant elastin for biomedical applications-advances in molecular design, expression platforms, and purification strategies: a review. Int. J. Biol. Macromol. 334 (Pt 2), 149116. 10.1016/j.ijbiomac.2025.149116 41271050

