# Supplementary material for: Construction and expression of recombinant human elastin with biological activities: effect of wound healing in vitro and in vivo
Source: Front Bioeng Biotechnol. 2026 Sep 10;14:1913985. doi: 10.3389/fbioe.2026.1913985 (PMC13601249; doi:10.3389/fbioe.2026.1913985)
Supplement: Supplementary file 1 [file Image1.pdf]

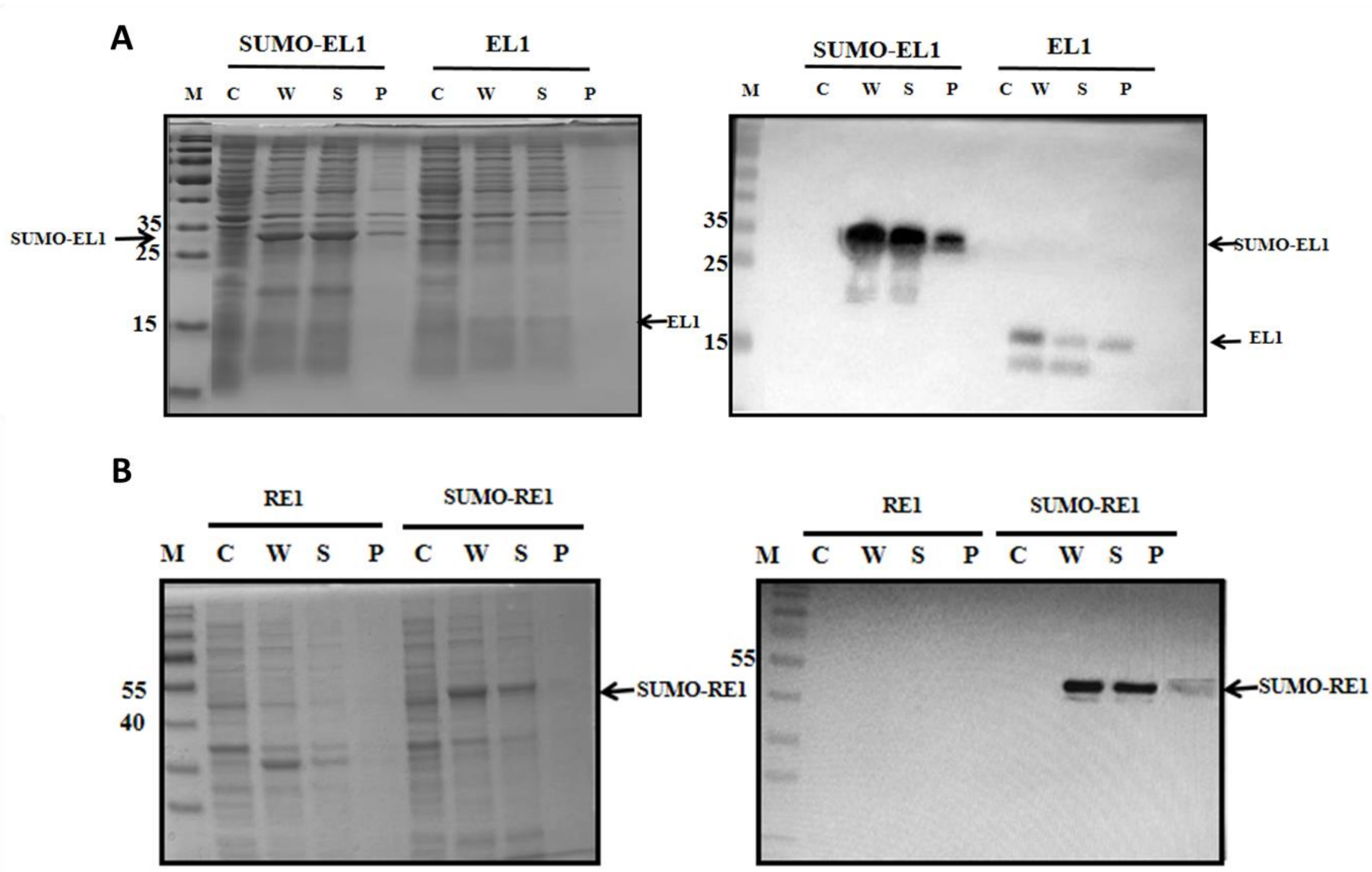

Figure. S1. Expression of fusion protein SUMO-EL1 and SUMO-RE1. (A) SDS-PAGE and Western blot (His-tag) analysis of SUMO-EL1 expression. (B) SDS-PAGE and Western blot (His-tag) analysis of SUMO-RE1 expression. M: Marker; C: Uninduced whole bacteria, W: Induced whole bacteria, S: Induced supernatant, P: Induced intracellular precipitates.

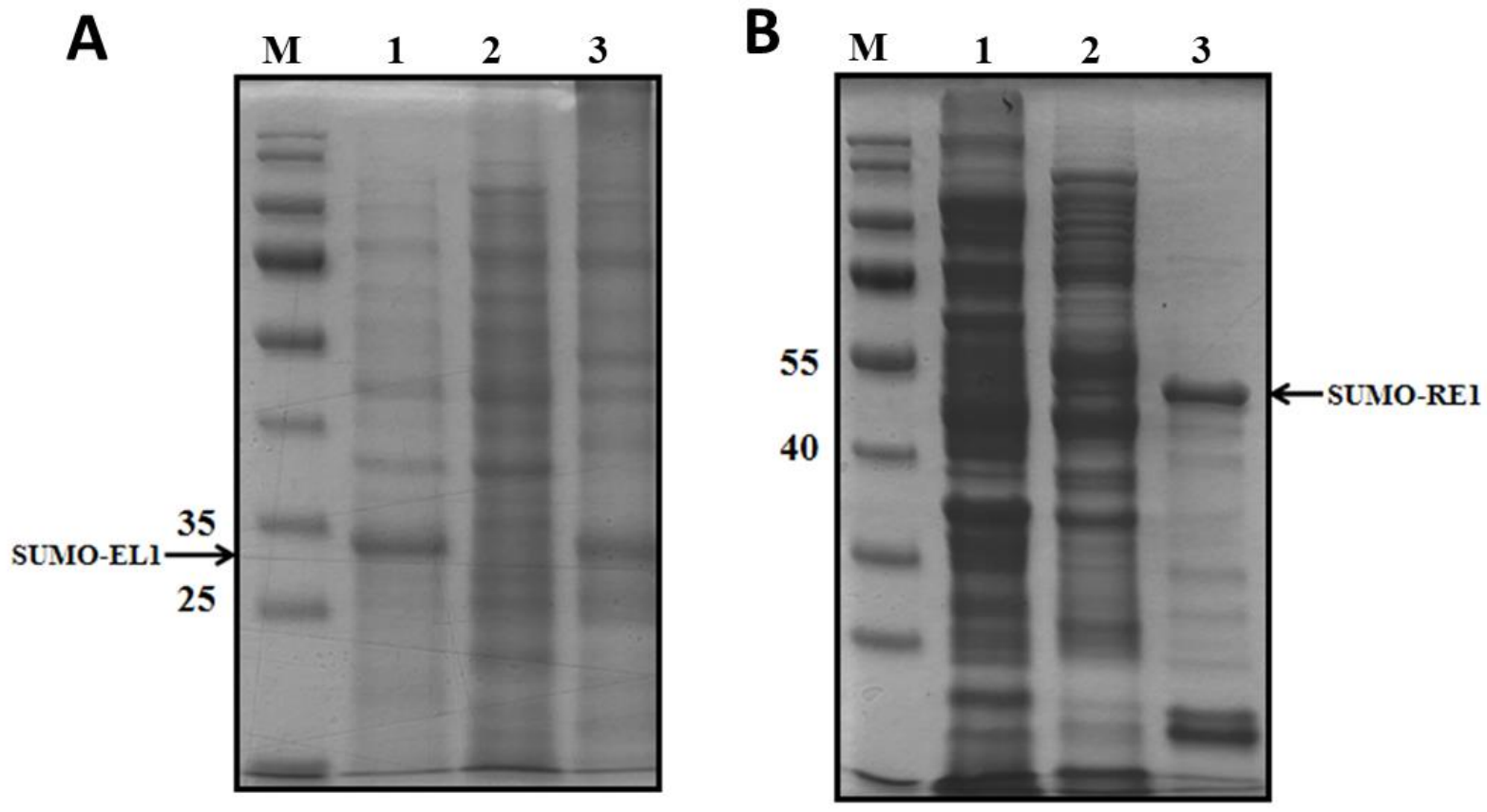

Figure. S2. Purification of fusion protein. (A) Ni column by affinity chromatography of SUMO-EL1 fusion protein. M: Marker; 1: cell lysate supernatant; 2: flow-through; 3: protein eluted by 60 mM imidazole. (B) Ni column by affinity chromatography of SUMO-RE1 fusion protein. M: Marker; 1: flow-through; 2: protein eluted by 60 mM imidazole; 3: protein eluted by 250 mM imidazole.

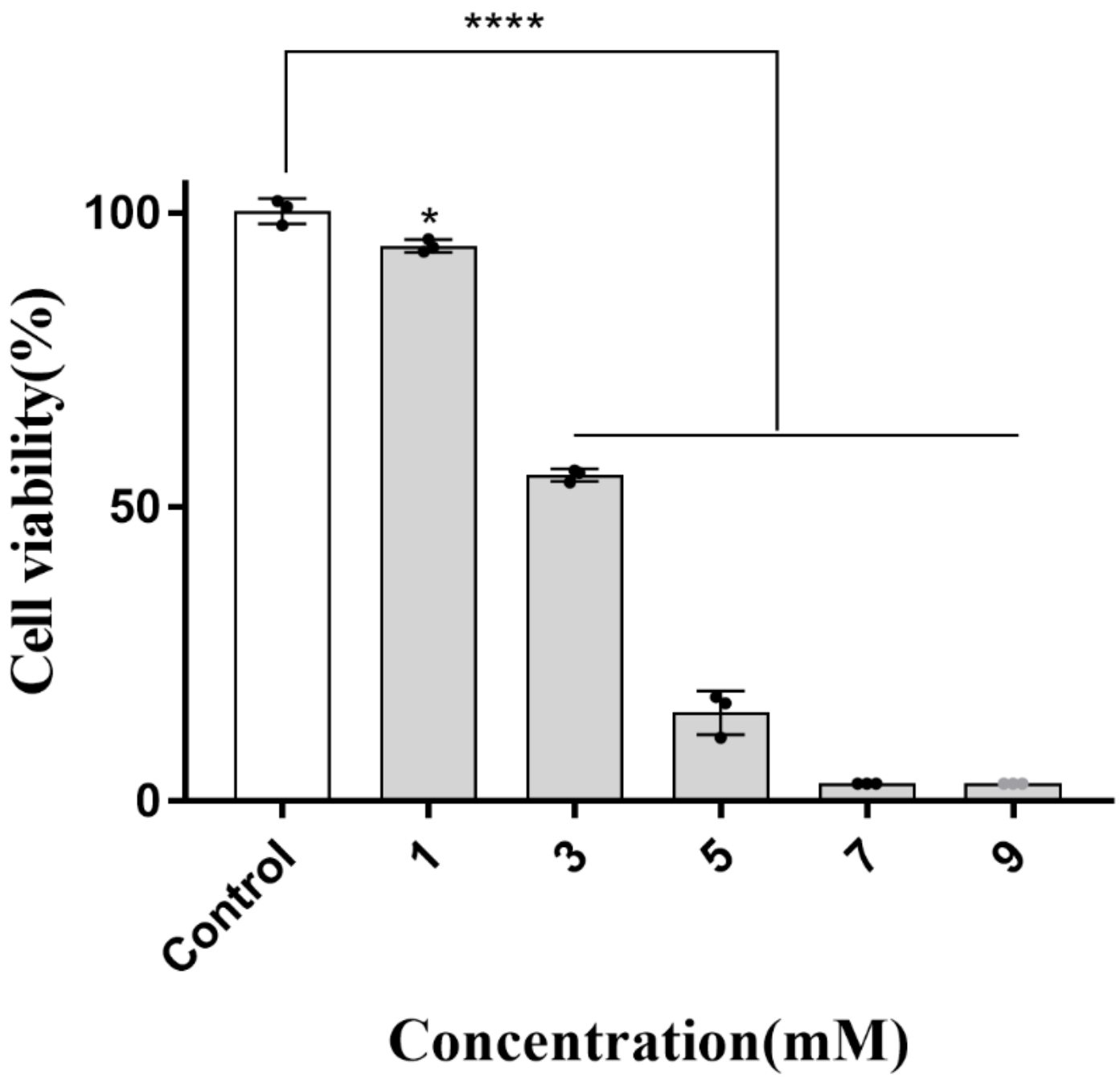

Figure. S3. 3T3 cells were treated with H_2O_2 of different concentrations for 24 hours.
